# g you The direct determination of haplotypes from extended regions of genomic DNA

**DOI:** 10.1186/1471-2164-11-223

**Published:** 2010-04-06

**Authors:** David Stirling, Michael J Stear

**Affiliations:** 1 Department of Haematology, Royal Infirmary of Edinburgh, Little France Crescent, Edinburgh EH16 4SA, UK; 2 Division of Animal Production and Public Health, Faculty of Veterinary Medicine, Glasgow University, Bearsden Road, Glasgow G61 1QH, UK

## Abstract

**Background:**

One of the major obstacles to the exploitation of genetic variation in human medicine, veterinary medicine, and animal breeding is the difficulty in defining haplotypes in unrelated individuals.

**Results:**

We have developed a Multiplex Double Amplification Refractory Mutation System combined with Solid Phase PCR on Fluorescently labelled beads. The process is inherently amenable to automation. It provides a high degree of internal Quality Control, as each PCR product is represented in duplicate on the bead array, and each SNP is tested against multiple partners. This technique can resolve very complex genotypes into their constituent haplotypes; it defined all the alleles at 60 SNP in exon 2 of the ovine *DRB1 *MHC locus in a sample of 109 rams. These 60 SNP formed 33 *DRB1 *exon 2 alleles; two of which had not been previously identified; although both of them have been independently confirmed.

**Conclusion:**

This technique has the same resolution as allele specific sequencing. Sequencing has the advantage of identifying novel polymorphic sites but where all SNP sites have been identified this novel procedure can resolve all alleles and haplotypes and identify novel combinations of polymorphisms. This method is similar in price to direct sequencing and provides a low cost system for direct haplotyping of extended DNA sequences.

## Background

One of the major priorities in genome analysis is to determine the joint inheritance and the population-wide associations among contiguous alleles on the same chromosome. This is known as the haplotype structure of a population and it is an essential part of the genetic architecture. The haplotype structure determines the usefulness of closely linked markers in identifying disease-susceptible individuals or genetically superior livestock. It also influences genetic interactions. For example, a polymorphism in a promoter, an enhancer or another regulatory element may interact differently with an allele on the same or on an opposing chromosomal strand.

Either deductive or direct approaches can be used to define haplotypes. The deductive approach infers haplotypes from the inheritance patterns in families or from the observed associations among genotypes [1]. This approach is widely used. However, the algorithms can have error rates between 2 and 48% [1,2].

Direct approaches analyse single chromosomes. The first techniques used cloning and sequencing [3], but this is time-consuming and impractical for large populations. Subsequently, SNP were used to design allele specific primers and the resulting products were then sequenced. This approach was used to haplotype a 6.4 Kb fragment spanning the APOE gene [4] and a 19 Kb portion of the human glucocorticoid receptor [5]. Lo et al [6] used allele-specific forward and reverse primers to haplotype two SNP's <500 bp apart. This work was further developed by Eitan and Kashi [7] who determined the haplotypes of 14 SNP in a 653 bp segment of the chicken HSP 108 gene. While this method allows long regions of DNA to be haplotyped, it is a two stage process, requiring initial sequencing or genotyping to define suitablele SNP that can be used for allele specific PCR. Unfortunately, the fragments that can be analyzed are of limited size, and the dependence on electrophoresis as the end point greatly limits sample throughput. Clearly, a simple, reliable and rapid method for direct haplotyping is urgently needed.

The approach that we have developed has two stages. In the first stage, PCR is performed in the presence of all forward and reverse primers both in solution and linked to the beads. This overcomes some of the difficulties of solid phase PCR [8,9] by allowing in-solution amplification of products, which are subsequently 'kidnapped' to the solid phase. In the second stage, the beads are washed free of non-covalently linked DNA, interrogated by allele specific primer extension with individual fluorescently labeled primers, washed and analyzed. This is a fully automatable procedure with a high degree of internal quality control.

We chose to test this procedure on the *DRB1 *locus of the sheep major histocompatibility complex. This locus and its homologues is among the most polymorphic in the mammalian genome. In sheep, over 100 alleles have been identified at this locus but additional alleles are believed to exist. The combination of high levels of diversity with unknown alleles provided a rigorous test of our procedure. In addition, sheep are an important commercial species and the *DRB1 *locus is associated with resistance to nematode infection [10,11], which is a major disease of livestock including sheep [12]. Therefore any results would have both scientific and commercial relevance.

## Results

**Figure 1:**
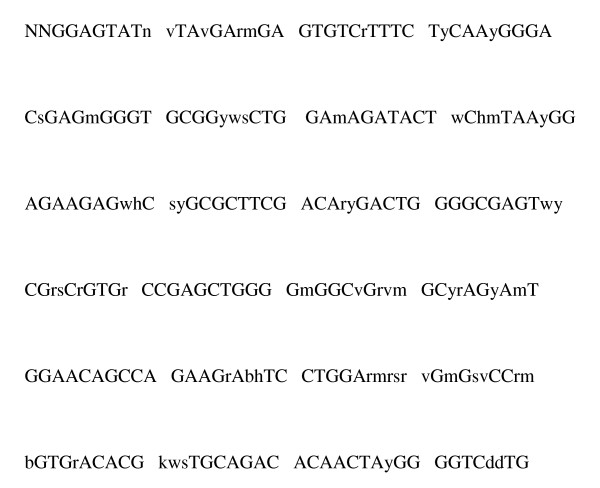
**Consensus sequence for reported alleles**.

Of the 237 nucleotides examined in exon 2 at the *DRB1 *locus, 60 (28%) were polymorphic. The majority of polymorphic sites were biallelic with only two nucleotides, but a substantial proportion had three or even four different nucleotides (Figure 1).

The direct haplotyping procedure identified 33 exon 2 alleles at the *DRB1 *locus in 109 Scottish Blackface Rams. PCR using allele-specific primers and sequencing gave identical results. Both techniques identified one or two exon 2 alleles in all animals. Therefore both techniques appeared to identify all alleles in the population.

The majority of these exon 2 alleles had not been previously identified in this breed of sheep, while two exon 2 alleles had not been previously identified in sheep of any breed. They were assigned Genebank accession numbers FJ213447 and FJ213448. These new alleles both represented novel combinations of nucleotides previously reported to be polymorphic. Both exon 2 alleles have been independently confirmed (Table 1).

**Table 1:** Alleles identified in Scottish Blackface sheep.

Allele	Frequency	Number	IPD name
Y10248	23.1	01	DRB1* 0101
AB017204	18.1	02	0901
U00216	10.2	03	0501
AB017218	6.5	04	1201
AB017230	5.1	05	0302
AF036561	4.6	06	0308
U00206	4.2	07	0102
AB061323	3.7	08	0301
AF036562	2.3	09	0304
AB017206	2.3	10	1101
FJ213447	2.3	11	0802
Y10245	1.9	12	2201
AF126441	1.4	13	N/A
AB017210	1.4	14	N/A
AB017228	1.4	15	N/A
U00212	0.9	16	N/A
FJ213448	0.9	17	1002
Z92728	0.9	18	N/A
Z92726	0.9	19	N/A
U00219	0.9	20	N/A
AB061372	0.9	21	0801
AB017212	0.9	22	1301
U00235	0.5	23	N/A
U00215	0.5	24	N/A
U00209	0.5	25	N/A
U00204	0.5	26	N/A
AY227049	0.5	27	1401
AF126434	0.5	28	N/A
AF036560	0.5	29	N/A
AB017226	0.5	30	1601
AB017216	0.5	31	N/A
AB017214	0.5	32	0402
AB017211	0.5	33	N/A

The allele assignments were made by the Immunopolymorphism database[21]. N/A: no assignment exists as yet (01/11/09). The allele names are the GenBank accession numbers. In several instances, there were several identical alleles with different accession numbers in the genbank database; in this case one accession number was arbitrarily chosen for clarity.

**Figure 2:**
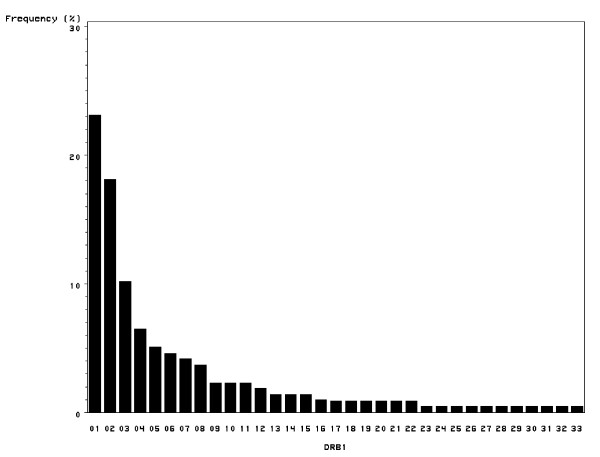
**Frequency chart of the 33 alleles in 109 Scottish Blackface rams**.

The names of the exon 2 alleles are listed in Table 1 while the frequencies are presented in figure 2. The distribution of exon 2 allele frequencies was very skewed. Three exon 2 alleles (DRB1*0101, *0501 and *0901: Y10248, U00216 and AB017204) accounted for more than 50% of the observations and the vast majority of the homozygosity observed. In contrast, 7 exon 2 alleles occurred in only 2 sheep while 11 exon 2 alleles were present in just one animal.

The fluorescent signal from the amplified products was readily distinguished from the background. The mean number of positive events in the absence of the SNP allele across all primers was only 9 ± 5. In contrast, the number of positive events in the presence of the SNP allele was 195 ± 12. Non-specific amplification was readily distinguished from allele-specific amplification by setting a threshold of 100 events.

## Discussion

A Multiplex double ARMS reaction on solid phase (Trans-Cistor) was used to determine the linkage phase of each pair of polymorphic sites and to resolve complex genotypes into constituent haplotypes. Essentially this technique determines whether the alleles at two linked SNP loci are in a trans or cis relationship. (Trans-Cistor). This approach has the same resolution as allele specific sequencing. In addition, unlike direct sequence based genotyping, it does not require the time-consuming and error prone assignment of alleles from a single polymorphic sequence. However, direct sequencing does have the advantage of identifying novel polymorphic nucleotides but for loci such as the MHC, the vast bulk of the variation is the result of novel combinations of polymorphisms at well-described positions. We have demonstrated that this technique (Trans-Cistor) is capable of identifying such novel exon 2 alleles. Importantly, both of the novel exon 2 alleles have been independently confirmed.

An alternative bead-based method has been developed independently [13]. In this procedure, oligonucleotides specific for SNP allele are attached to differently-coloured fluorescent beads and use to probe amplicons which have been generated from the test genome. This procedure relies on allele-specific hydridisation and like the double ARMS procedure described here allows high-throughput determination of SNP haplotypes.

ARMS based techniques can be prone to both false positive (due to promiscuous priming) and false negative results (due to PCR failure). VOSS addresses these potential problems in a number of ways. Each PCR reaction uses at least four specific primers. When the sample is heterozygous at both SNP pairs, four positive reaction products are produced. Heterozygosity at only one of the pair produces 2 reaction products, whereas homozygosity at both members of the pair produces a single product. Each SNP is tested multiple times within the same reaction. In addition, there is a minimum of two positive fluorescent bead populations for every expected reaction. This inherent quality control greatly aids the confident assignment of haplotype. Such built in redundancy might generally be thought to be expensive. However the cost of the fluorescent beads (£1.60 or $3.00 per test) is in the same order as single sequencing reactions, and with all other reagents and consumables being common to both techniques, Trans-Cistor compares favorably in economic terms. Moreover, it is the potential to automate from PCR set up to haplotype identification that shows the greatest potential advantage.

Solid phase PCR has been reported to have an efficiency less than half that of liquid phase [14]. We have adopted two strategies to address this. As each reaction is represented in both the liquid and solid phase, there is always a more efficient liquid phase reaction, producing template for the solid phase reaction. Additionally, the use of laser detection of fluorescent product on beads, allows detection of extremely small quantities of DNA, far less than could be visualized with ethidium bromide stained electrophoresis gels. Furthermore, while multiplex PCR reactions are prone to produce many spurious products that cannot be easily identified by electrophoresis, the detection system used here only identifies those products with the expected priming site at each end, eliminating the 'multiplex noise'.

PCR based techniques are always limited by the size of the DNA region which can be amplified efficiently and this may be a specific limitation on Solid phase PCR. We have tested this by amplifying portions of exon 14 of the human clotting factor VIII gene. Solid phase amplicons up to 10 Kb could routinely be produced, and amplicons up to 3.5 Kb could be readily produced in multiplex reactions (data not shown), demonstrating the power of the technique to determine haplotype over extended regions.

The double ARMS [6] method is a general approach to determining linkage phase of polymorphic markers. Trans-Cistor offers the ability to automate all the way through to haplotype. Its application extends to all uses of haplotype information including linkage analysis for the diagnosis of genetic diseases. Population studies on the geographical distribution of haplotypes can be performed without pedigree analysis. Haplotype information over extended regions may also be used to study genetic recombination.

The solid phase double ARMS is a robust method that has been applied to the complex DRB1 locus in sheep. However, this method could be used routinely for haplotype determination and could have many diagnostic applications. Potential applications include genotyping of viral strains in mixed infection, chimaerism following bone marrow or stem cell transplantation, and mapping the boundaries of translocations in malignant cell clones.

The Trans-Cistor procedure improves the accuracy with which linkage disequilibrium can be estimated. We compared linkage disequilibrium between SNP at positions 8 and 233. The first method used in individuals whose haplotypes are unknown, uses a composite linkage disequilibrium coefficient [15]. In our sample the most common haplotype was AA with a frequency of 0.31 and a correlation coefficient of 0.23. In contrast, when the directly determined haplotypes were used the correlation coefficient was 0.17. As linkage disequilibrium is widely used to identify genetic markers for disease, the ability to obtain correct estimates in unrelated individuals without testing extended families will be invaluable.

## Conclusion

The solid phase double ARMS approach combines exquisite sensitivity with the specificity to detect a minority DNA population. High accuracy results from multiple testing of each SNP and the repeated checking of phase against multiple sites.

## Methods

### Animals

We examined 109 rams. All rams were purebred Scottish Blackface rams from 12 Scottish farms in the 'Blackface Elite Sire Reference Scheme'. This is the largest sheep breeding scheme in the UK. Twenty-two different farms have the same breeding objectives and they share and exchange rams every year to facilitate comparisons among lambs from different farms. Over 10,000 lambs are produced every year. Blood sampling was covered by Home Office personal and project licences to M. J. Stear.

### Reagents

Primers for PCR amplification were purchased from MWG (Ebersberg, Germany), Expand long range Taq DNA polymerase from Roche Diagnostics (Burgess Hill, UK) and GoTaq Taq polymerase and dNTPs from Promega (Southampton, UK). Multiplex PCR Buffer was obtained from Point-2-Point Genomics (Edinburgh UK). Luminex beads were purchased from BioRad (Hemel Hempstead, UK). Standard reagents were from SigmaAldrich (Gillingham, UK) or BDH Laboratory Supplies (Poole, UK) in analysis grade, if not otherwise stated.

### Assay design

**Figure 3:**
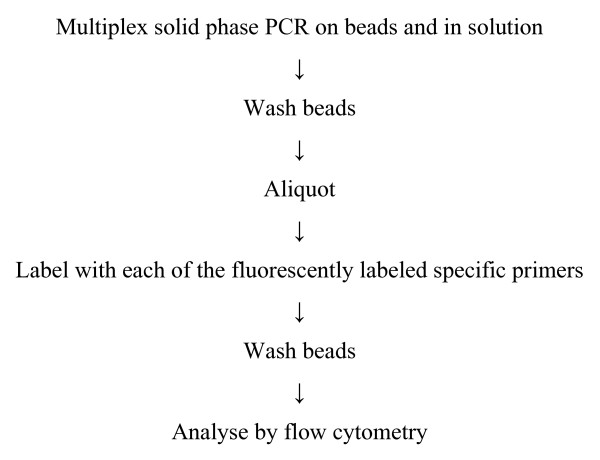
**A short summary of the haplotyping procedure**.

**Figure 4:**
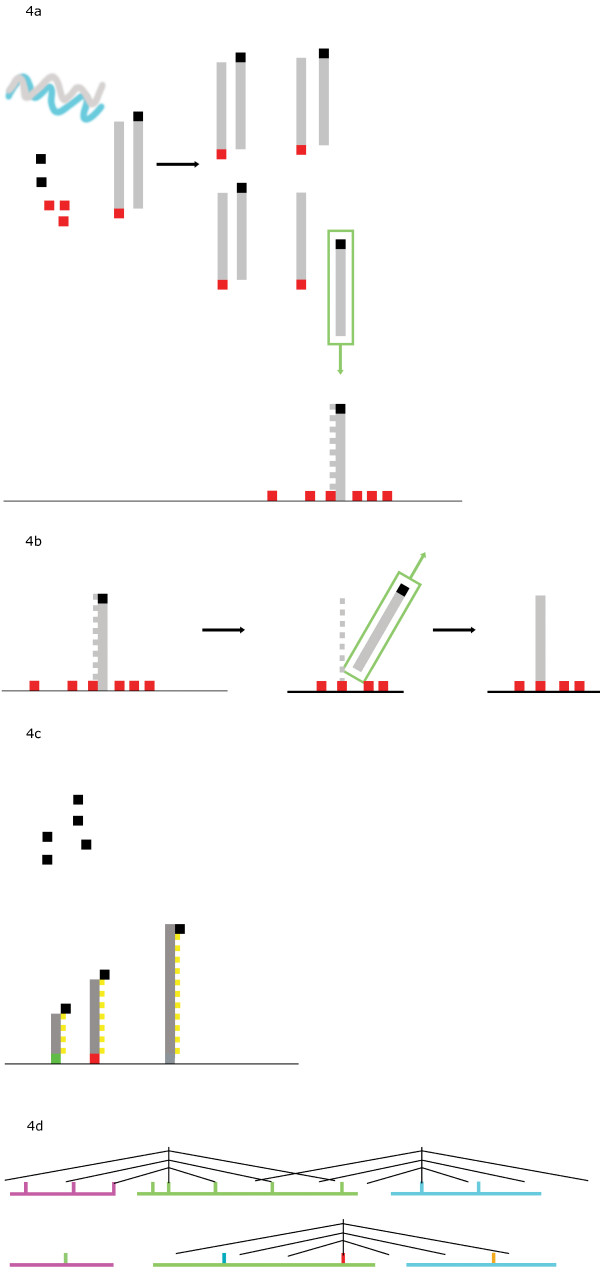
**Diagram of the typing procedure**. In 4A, in the first phase, allele specific primers are shown in red and black. The sample is shown as wavy lines in grey and blue. There is a PCR reaction in the supernatant with forward and reverse allele-specific primers. The PCR amplicon is then kidnapped and attaches to one of the reverse primers on the solid phase. In the presence of nucleotides and polymerase a second chain is formed. In 4B, the kidnapped amplicon, which is only held by hydrogen bonding, is released by heating. Washing leaves a covalently bonded single strand. In 4C, a reverse primer is introduced which binds to the covalently attached amplicon. In reality we have all possible forward and reverse primers illustrated as 3 candidate SNP in a row in green, red and grey. In 4D, the results from the separate reactions can be combined to indicate the SNP haplotypes.

The assay is a multiplex, double Double Amplification Refractory Mutation System (ARMS) based haplotyping method (ARMS [16], also known as PCR amplification of specific alleles or PASA). A short summary of the procedure has been provided (Figure 3) and a diagram illustrating the procedure in Figure 4. Fundamental to this approach is the efficient amplification of product on a defined position on a solid support. Here we have used fluorescently tagged beads, although a conventional microarray could also be used. Up to 100 separately anchored PCR reactions can be multiplexed in the same tube. The assay was developed as a two-stage process. In the first stage, all oligos (forward and reverse) involved in the multiplex reaction are present in the solution phase, and also bound to beads. Thus the solution phase reaction is able to amplify sequences, which then act as templates for the solid phase reaction. After this first stage, the beads are washed, and aliquots of the bead mixture labeled by one round of primer extension with each of the individual fluorescently labeled oligonucleotides. On analysis, products can be detected from every possible combination of forward and reverse oligonucleotides, thus directly identifying the phase of each polymorphism. This process (Trans-Cistor) is covered by patents held by Point-2-Point Genomics: UK & Au patent no 2377220, European Patent EP137 3566, International PCT WO 02081743.

### Primer design

Primer sequences are listed in Table 2. These were designed with reference to an alignment of the known allele sequences for ovine DBR1 (figure 1). Primers were selected to avoid internal or paired complementarities, and to have melting temperatures of 55°C ± 3°C. For each primer position, oligonucleotides were synthesized, to offer allelic discrimination at the 3' terminal base. Where polymorphic bases were included other than at the extreme 3' end of the sequences these were either synthesized as 'wobbles', or used as part of the allelic discrimination set (see Table 2 legend). For immobilization to beads, oligonucleotides were synthesized with a 5' amino link C12 spacer to minimize steric hindrance [9,17].

**Table 2:** Sequences of allele specific primers used in this study.

Primer	FORWARD	Position in Reference sequence
F1 c/t	GARMGAGTGTCRTTTCT**Y**	13 - 30

F2 c/t	GAGTGTCRTTTCTYAAA**Y**	17- 34

F3 g/c	TTTCTYAAAYGGGAC**S**	25 - 40

F4 ac	GGACSGAG**A**GGGTGCGG**C**	35 - 53

F5 ct	GGACSGAG**C**GGGTGCGG**T**	35 - 53

F6 a/t	GACSGAGMGGGTGCGGY**W**	36 - 54

F6 a/c	GGGTGCGGYWSCTGGA**M**	45 - 61

F7 a/t/c	CTGGAMAGATACTWC**H**	56 - 71

F8 a/t	HMTAAYGGAGAAGAG**W**	71 - 86

F9 a/t/c	HMTAAYGGAGAAGAGW**H**	71 - 87

F10 a/g	GACTGGGGCGAGTWYCG**R**	104 - 121

F11 g/c	TGGAACAGCCAGAAGGGA**S**	158 - 176

		

	**REVERSE**	

R1 g/a/t	CTTCTGGCTGTTCCA**D**	172 - 157

R2 a/g	CTTCTGGCTGTTCCADT**R**	172 - 155

R3 c/t	CTGGCTGTTCCADTRCT**Y**	169 -152

R4 ct	CTGTTCCADTRGTYRGCK**CT**	165 - 146

R5 tc	CTGTTCCADTRGTYRGCK**TC**	165 - 146

R6 g/a/c	CTGCAGTACGTGTCCAC**V**	216 - 199

Allele specificity is conferred by the bases in bold upper case. All primer sequences are shown 5'-3'.

### Conjugation to Beads

Luminex (xMAP) suspension array technology is based on polystyrene beads with a diameter of 5.6 μm that are internally dyed with various ratios of two spectrally distinct fluorophores. Thus, an array of 100 different bead sets with specific fluorescent ratios is created. These sets are combined to a suspension array and, due to their unique fluorescence pattern, allow up to 100 different analytes to be measured simultaneously in a single reaction. Conjugation of oligonucleotides to carboxylated microspheres was done by a minor modification of the Luminex protocol [18,19]. Briefly, 5 × 10^6 ^unlabeled, carboxylated microspheres, were vortexed in a 1.5 ml microcentrifuge tube, dispersed by sonication for 30 s, centrifuged at 8000 g for 1 min and, after supernatant removal, adjusted to 0.1 M 2-(N-morpholino) ethanesulfonic acid, pH 4.5. An aliquot of 1 nmol 5' amino-modified oligonucleotide was added, followed by 2.5 μl of fresh 1-ethyl-3-(3-dimethyaminopropyl)carbodiimide-HCl (Perbio Science, Cramlington UK) from a 10 mg/ml stock. After 1 h at room temperature the microspheres were repeatedly washed by centrifugation and stored at 4°C in the dark. A control oligonucleotide, with 5' amino link C12 spacer and 3'fluorescent label was conjugated with each batch of reactions to estimate conjugation efficiency.

### Phase-1 reaction conditions

Reaction conditions, with MgCl_2 _concentration, primer concentrations, annealing temperature, and additives (e.g., bovine serum albumin and DMSO, TEMAC) were tested extensively to optimize the PCR reactions. Optimal conditions were 1× Multiplex PCR buffer, 2 nM MgCl_2_, 200 μM of each dNTP, 25 mU/μL of GoTaq DNA Polymerase, and sterile deionized water to the appropriate volume. Optimum primer concentrations were determined empirically. Each reaction included 2,000 beads of each specificity. After an initial denaturation at 95°C for 3 min, there were 10 cycles of 95°C for 15 s, 60°C for 20 s, 70°C for 45 s, and 20 cycles of 10 cycles of 95°C for 15 s, 55°C for 20 s, 70°C for 45 s. On completion of thermal cycling, the beads were washed 3 times by dilution in 200 μl TE (10 mM Tris, pH 7.4; 1 mM EDTA) and 30 s incubation at 95°C.

### Phase-2 reaction conditions

For the second phase reaction, the beads were resuspended in a 25 μL reaction volume containing 1× Multiplex PCR buffer, 1 nM MgCl_2_, 50 μM of each dNTP, 25 mU/μL of GoTaq DNA Polymerase and 0.05 μM specific labeled primer. The reaction mixture was then incubated at 95°C for 15 s, and 65°C for 30 s. Beads were subsequently washed 3 times by dilution in 1× Multiplex PCR buffer and 30 s incubation at 75°C.

### Analysis of beads

Following the final wash, the beads were resuspended in 0.5 ml TE, and analysed by flow cytometry on a FACSCalibur Flow cytometer (BD Biosciences). A single bead population was gated on forward and side scatter. 100,000 events from this population were then analysed on FL3 and FL4 to discriminate each of the bead populations. These individual bead populations were then gated, and analysed for FL1 and FL2 for the reporter fluorescence. A positive fluorescence was defined as being at least four fold higher than background fluorescence (measured in un-reacted beads). For a reaction to be scored as positive, both the beads representing that reaction (ie with the forward or reverse primer in the solid phase) had to be positive.

### PCR of Sheep DNA for sequencing

DNA was extracted from jugular blood samples from 109 sheep, using standard phenol/chloroform methods. The second exon of *DRB1 *was amplified using a semi-nested PCR technique. The first round of PCR was performed with primers ERB3 (5'-GGAATTCCTCTCTCTGCAGCACATTTCCT-3') and HL031 (5'-TTTAAATTC GCGCTCACCTCGCCGCT-3') [20]. 100 ng of genomic DNA was amplified by PCR in a total volume of 25 μl of PCR buffer (1× multiplex buffer, 1.5 mM MgCl_2_, and 120 μM dNTP), to which 0.2 mM each primer, and 25 mU/μL of GoTaq DNA Polymerase, and sterile deionized water to the appropriate volume had been added. Reactions were performed under the following conditions: 5 min at 94°C, followed by 15 cycles of 94°C for 15 s, 50°C for 30 s, and 72°C for 60 s, with final extension at 72°C for 5 min. We amplified 3 μl of the resulting mixture with primers ERB3 and SRB3 (5'-AAGTCGACCGCTGCACAGTGAAACTC-3') for the second round of PCR. The conditions for the second round of PCR were one cycle for 5 min at 94°C, followed by 30 cycles of 94°C for 30 s, 60°C for 30 s, and 72°C for 60 s with final extension at 72°C for 10 min. PCR products were checked by agarose gel electrophoresis, before being sequenced with primers ERB3 and SRB3. Forward and reverse sequences were aligned with Sequence Navigator Software (ABI), and where heterozygous bases were identified, a third sequencing reaction using an appropriate allele specific oligonucleotide (Table 2) was performed. Analysis of all sequences allowed haplotypes to be assigned.

### Nomenclature

Conventionally, a locus is the site of a gene on a chromosome while an allele is an alternative form of a gene and a haplotype represents a contiguous set of genes on a chromosome. The size of a gene can vary from a single nucleotide in an SNP allele to over 150,000 nucleotides in the Huntington's Disease locus. Some loci such as the MHC class II genes contain many SNP even within a single exon. Therefore a protein-coding allele can also be considered as an SNP haplotype. Usually, the context makes matters clear but to minimize potential confusion, we occasionally refer to SNP alleles, SNP haplotypes and protein-coding alleles.

## Competing interests

The authors declare that they have no competing interests.

## Authors' contributions

DS carried out the molecular genetic studies and drafted the manuscript. MJS participated in the design of the study, carried out statistical analyses and revised the initial draft. Both authors read and approved the final manuscript.
